# Testosterone Trajectories and Reference Ranges in a Large Longitudinal Sample of Male Adolescents

**DOI:** 10.1371/journal.pone.0108838

**Published:** 2014-09-30

**Authors:** Ammar Khairullah, Laura Cousino Klein, Suzanne M. Ingle, Margaret T. May, Courtney A. Whetzel, Elizabeth J. Susman, Tomáš Paus

**Affiliations:** 1 Rotman Research Institute and Institute of Medical Science, University of Toronto, Toronto, Canada; 2 Department of Biobehavioral Health and Penn State Institute of the Neurosciences, The Pennsylvania State University, State College, Pennsylvania, United States of America; 3 School of Social and Community Medicine, University of Bristol, Bristol, United Kingdom; 4 Department of Biobehavioral Health and the Center for Healthy Aging, The Pennsylvania State University, State College, Pennsylvania, United States of America; 5 Department of Biobehavioral Health, The Pennsylvania State University, State College, Pennsylvania, United States of America; Brock University, Canada

## Abstract

**Purpose:**

Pubertal dynamics plays an important role in physical and psychological development of children and adolescents. We aim to provide reference ranges of plasma testosterone in a large longitudinal sample. Furthermore, we describe a measure of testosterone trajectories during adolescence that can be used in future investigations of development.

**Methods:**

We carried out longitudinal measurements of plasma testosterone in 2,216 samples obtained from 513 males (9 to 17 years of age) from the Avon Longitudinal Study of Parents and Children. We used integration of a model fitted to each participant’s testosterone trajectory to calculate a measure of average exposure to testosterone over adolescence. We pooled these data with corresponding values reported in the literature to provide a reference range of testosterone levels in males between the ages of 6 and 19 years.

**Results:**

The average values of total testosterone in the ALSPAC sample range from 0.82 nmol/L (Standard Deviation [SD]: 0.09) at 9 years of age to 16.5 (SD: 2.65) nmol/L at 17 years of age; these values are congruent with other reports in the literature. The average exposure to testosterone is associated with different features of testosterone trajectories such as Peak Testosterone Change, Age at Peak Testosterone Change, and Testosterone at 17 years of age as well as the timing of the growth spurt during puberty.

**Conclusions:**

The average exposure to testosterone is a useful measure for future investigations using testosterone trajectories to examine pubertal dynamics.

## Introduction

Puberty (activation of the hypothalamic-pituitary-gonadal axis) and adolescence (maturation of adult social and cognitive behaviours) are intertwined [1]. Variations in pubertal trajectories have been associated with those in physical and behavioral development [2]. For example, an earlier onset of puberty predicts a higher body mass index and central fat-mass in 19-year old men [3]. Early pubertal timing and faster pubertal tempo appear to be associated with depressive symptoms in male adolescents [4].

The activation of the hypothalamic-pituitary-gonadal (HPG) axis stimulates the male testes and, in turn, increases production of testosterone [5], [6]. In males, testosterone is a sex steroid that plays a critical role in numerous pubertal processes, including genital growth [7], change in body composition [8], and maturation of the brain [9], [10]. Given testosterone’s crucial role in puberty, its dynamics may be driving associations previously ascribed to pubertal timing and tempo [4], thus underscoring the importance of studying testosterone trajectories in relation to healthy and abnormal development.

A number of human studies have investigated changes in testosterone levels throughout the lifespan of males. We reviewed the literature for reports that provided normal ranges of total testosterone during male adolescence, pooled together data from the five studies identified [11]–[15], and report the weighted values. Of the five studies included, one (Schnakenburg, 1980) used plasma and the other four studies used serum to quantify testosterone levels. The ethnicity of participants was often not described. One of the studies was conducted in the United States (Lee, 1974), and the other four in Europe. Longitudinal measures of testosterone are a pre-requisite for investigating testosterone trajectories in adolescent males. To this end, we quantified levels of total testosterone from longitudinal samples in typically developing males from the Avon Longitudinal Study of Parents and Children (ALSPAC). We also include ranges for sex hormone binding globulin (SHBG) and the calculated free and bioavailable fractions of testosterone, as these are of interest when studying the effect of testosterone on the brain and body. The total testosterone describes the amount of hormone produced by the body whereas the bioavailable testosterone refers to the amount of testosterone capable of crossing cell membranes [16]. Finally, we describe a measure of average exposure to testosterone throughout adolescence derived from participants’ testosterone trajectories, and examine the trajectories in context of the adolescent growth spurt, as indexed by Age at Peak Height Velocity (APHV), and several characteristics of the testosterone trajectories.

## Methods

### The Avon Longitudinal Study of Parents and Children

We used blood samples obtained from participants in the Avon Longitudinal Study of Parents and Children (ALSPAC), a birth cohort that recruited 14,541 pregnant women resident in Avon, United Kingdom with an expected delivery date between April 1991 and December 1992. Since then, the children and their parents have been studied extensively with longitudinal data available from self-administered questionnaires sent to their homes, linkages to medical records, and through clinical examinations carried out during study visits [17]. Ethical approval for ALSPAC was obtained from the ALSPAC Ethics and Law Committee and the Local Research Ethics Committees. Data used for this submission will be made available on request to the ALSPAC executive committee (alspac-exec@bristol.ac.uk). The ALSPAC data management plan describes in detail the policy regarding data sharing, which is through a system of managed open access (http://www.bristol.ac.uk/alspac/researchers/data-access/documents/alspac-data-management-plan.pdf).

Blood samples were taken at select visits over the course of the study, roughly at the following years of age: 7, 9, 11, 13, 15, and 17. We obtained and analyzed sex steroids from the blood samples of a subset of 513 male participants who had been recruited for further study using magnetic resonance imaging (MRI). We did not utilize blood samples from the 7-year visit, as most individuals are likely to have very low or undetectable levels of sex steroids at this age. All participants had a maximum of five and a minimum of three blood samples across these five visits (5 samples: n = 232; 4 samples: n = 213; 3 samples: n = 68).

### Quantification of Testosterone and Sex Hormone Binding Globulin

The blood samples, in the form of lithium heparin plasma, were sent from ALSPAC to the Biomarker Core Laboratory at The Pennsylvania State University, where enzyme-linked immunosorbent assays were conducted - using commercially available kits - to determine plasma concentrations of testosterone (EIA-1559; DRG International; Springfield, New Jersey, USA) and SHBG (IB79131; Immuno-Biological Laboratories, Inc.; Minnesota, MN, USA). For testosterone and SHBG, the sample test volumes were 25 ul and 10 ul, respectively. The assays had lower limits of sensitivity of 0.28 nmol/L (testosterone) and 0.77 nmol/L (SHBG), with average inter- and intra-assay coefficients of variation less than 10%. The assay sensitivity is calculated by subtracting two standard deviations from the mean of 20 identical runs of the zero standard.

For quality-control purposes, 10% of participant samples were tested in duplicate, balanced across plates while the rest of the samples were run in singlet to increase efficiency. All samples from a participant were run on the same plate. Of the samples run in duplicate, test values that varied by more than 5% were subject to repeat testing. The first-well values from all samples are used in data analyses for consistency. For testosterone samples that yielded undetectable hormone values or values below the lower limit of sensitivity for the assay (n = 199; 8.9% of all samples; 136 from the 9-year visit, 58 from the 11-year visit, and 5 from the 13-year visit), we used the lower bound value of the detectable range for the assay: 0.28 nmol/L.

### Effects of Age and Circadian Rhythm on Testosterone

Since testosterone demonstrates a circadian rhythm [18] and the time of venipuncture was not standardized across study participants due to logistical reasons, we used multilevel modeling to predict total testosterone measurements at a standard time of day (12 PM). The effect of time of venipuncture on testosterone levels differed across age groups; therefore separate models were fitted for each visit (9, 11, 13, 15 and 17 years). In each of these models age was included as a continuous variable (note that age varied very little between participants at each visit – see Table 1) and time of sample was included as a categorical variable split into half-hour increments. The models fitted for ages 15 and 17 also included age squared, as there was evidence of a quadratic effect of age on testosterone. We assessed model fit by comparing the observed with the predicted values, and the best fitting models were chosen from which to obtain predicted values of testosterone. There were clear differences between observed and predicted values at age 13 indicating that models for age 13 did not fit well. We hypothesized that this was because age 13 is the time when puberty had begun in some but not all boys. We therefore fit the age 13 model separately for those who had and had not reached age at peak height velocity, which is a proxy for pubertal onset [19]. In these models, time of sample was again modeled as a categorical variable using half-hour increments. Age was not explicitly included in these models since participants were split based on whether they reached their age of peak height velocity, a variable that correlates with chronological age [19], [20].

**Table 1:** Hormone Values from the ALSPAC sample.

Age	n	Time ofVenipuncture	Total Testosterone(nmol/L)	Adjusted Total Testosterone*(nmol/L)	SHBG (nmol/L)	Free Testosterone(pmol/L)	Bio-available Testosterone(nmol/L)	% ReachedAPHV
9.82(.30);9.42–11.58	441	13.78(2.05); 10.83–16.87	.72(.58);.29–4.39	.82(.09);.73–1.39	92.28(43.64); 4.33–262.29	8.38(3.33); 2.72–30.92	.17(.07);.06–.64	1
11.69(.21);11.33–13.08	482	13.74(2.14); 10.75–19.38	1.47(1.83);.29–15.24	1.55(1.23);.08–10.71	74.90(37.45); 4.88–267.52	20.36(23.00); 0.97–193.54	.42(.47);.02–3.98	7
13.81(.17);13.08–14.67	414	13.81(2.13); 9.75–19.05	6.87(5.10);.29–35.53	9.05(4.66); 3.50–28.30	45.41(26.03); 3.53–164.24	186.86(125.34); 20.60–582.22	3.84(2.57);.42–11.96	69
15.38(.25);14.5–17.33	464	10.31(1.97); 8.00–14.00	15.18(5.48);.85–45.62	14.81(2.72); 7.40–29.82	30.81(13.32); 4.01–89.09	356.94(96.07); 81.86–638.33	7.33(1.97); 1.68–13.11	95
17.70(.33);16.58–19.17	413	10.66(1.99); 8.33–15.00	17.05(5.41); 1.63–42.48	16.50(2.65); 8.68–28.59	26.25(12.37); 3.35–140.09	438.81(96.12); 56.72–780.88	9.01(1.97); 1.17–16.04	100

Values are reported as Mean (SD); Range. Time of venipuncture is calculated as hour plus minutes/60. The data is taken from the following ALSPAC Focus Clinics (different visits): Focus at 9, Focus at 11, Teen Focus 2, Teen Focus 3, Teen Focus 4.

*Testosterone values are adjusted for age and time of venipuncture (see methods).

### Measure of Average Exposure to Testosterone Throughout Adolescence

We calculated a measure of average exposure to testosterone throughout adolescence for participants based on the area under the curve of their testosterone trajectory. The longitudinal measurements of total testosterone and the respective age in months at venipuncture were used to plot the participant’s total testosterone values during his childhood and adolescence. The shape preserving spline-interpolation function from the MATLAB Basic Fitting toolbox (Mathworks, Natick, MA USA) was used to generate a curve through the data-points. The area under the curve (integral) was calculated, with respect to ground, for the section of the spline between the first and last blood sample. This is similar to the method applied by Pruessner and colleagues in context of cortisol sampled at multiple points within an hour [21]. To accommodate variations between participants’ age ranges from the first to the last visit, we standardized this variable by dividing the integral value for each participant by their exact age range (in months) between the first and last visit. The resulting value is an estimate of the arithmetic mean of monthly measurements, if testosterone were sampled each month throughout the participant’s adolescence.

### Growth Spurt and Characteristics of Testosterone Trajectories

For each participant, we calculated Age at Peak Height Velocity (APHV) using up to nine height measurements collected at each ALSPAC visit, from 7 through 17 years of age, as described previously [19]. Briefly, the height data for each participant are fitted with a cubic spline from the Basic Fitting Toolbox in MATLAB (Mathworks, Natick, MA USA). Taking the derivative of the height curve gives us the growth curve, from which we ascertain the Peak Height Velocity and the corresponding age (in months) when this occurs.

We estimated the analogous measures for testosterone data, Peak Testosterone Change and Age at Peak Testosterone Change, by applying the same spline method. This calculation was restricted to participants who had blood collected at all five visits (n = 232), given that fewer than five data-points would likely lead to imprecise estimates.

### Free and Bioavailable Testosterone

To calculate levels of free testosterone and to derive values of bio-available testosterone, we used the equation (Total Testosterone = Free Testosterone+SHBG Bound Testosterone+Albumin Bound Testosterone) in conjunction with the (measured) SHBG and (estimated) albumin concentrations as described by Södergård [22].

### Statistics

We used Stata version 12.1 (StataCorp LP, College Station, TX) for multilevel modeling to adjust values of Total Testosterone. Other statistical analyses used JMP 9 for Macintosh (SAS Institute, Inc., Cary, NC): simple linear regression was used to relate the measure of average testosterone exposure to APHV, Age at Peak Testosterone Change, Peak Testosterone Change, and Total Testosterone at 17 years. In order to visualize participants’ testosterone trajectories and to investigate whether they varied in form between those with higher or lower average testosterone, we divided participants into quintiles based on the measure of average exposure to testosterone and graphed trajectories separately for each quintile.

### Review of Published Data

To compare our results with the published values of testosterone, we reviewed the literature and included studies that reported either a mean (with standard deviation) or a median (with range) for values of total testosterone measured from plasma or serum samples in males between the ages of 6 and 20 years. Note that the type of a sample (plasma vs. serum) may not affect measured values; we have conducted a pilot study using blood samples from 10 adult participants (5 female) to compare testosterone values measured in serum and plasma (sodium heparin, or lithium heparin, or EDTA) and found that these values showed correlations of >0.99 across the different media.

To identify such studies, we conducted a PubMed search in January 2012, using combinations of the following search terms: ‘testosterone’, ‘androgen’, ‘normal’, ‘reference range’, ‘adolescence’, ‘childhood’, ‘serum’, and ‘plasma’. We excluded studies for the following reasons: presenting data in a graph without an accompanying table, presenting data in subgroups of large age-ranges (greater than 2-year intervals), or reporting reference ranges for salivary testosterone or free testosterone without total testosterone. Furthermore, within the selected studies, we included only those age subgroups containing at least 10 participants.

First, we standardized the units for total testosterone into nanomoles per liter using a conversion factor where necessary (1 ng/mL = 3.467 nmol/L). For studies reporting median and range, we used methods described by Hozo and colleagues to estimate the mean and standard deviation (SD) [23]. When pooling data across studies for each age subgroup across studies, we used equations from Borenstein and colleagues to compute the weighted mean and corresponding standard deviation [24].

## Results

### ALSPAC: Comparing the full cohort and the subsample

The supplementary table (Table S1) shows a comparison between the subsample of 513 males included in this study and the rest of the ALSPAC males who survived to at least 1 year of age (n = 7024). The subsample differs from the full sample on a number of variables in a manner consistent with being from families with a higher parental education. Similar pattern of attrition have been observed in other studies [25].

### ALSPAC: Testosterone levels

The models we fitted to remove the effect of circadian rhythm explained 1.7%, 9.5%, 5.8%, and 6.3% of the variance in total testosterone at the 9, 11, 15 and 17-year visits, respectively. Models for the 13-year visit were fitted separately for participants who had not yet reached APHV and those that had passed APHV; the variance explained was 17.3% and 22.1%, respectively. Note that the relatively high proportion of variance in the unadjusted levels of testosterone during the 13-year old visit is due to the combination of rising testosterone levels and morning-afternoon sampling schedule in this visit (see Discussion for details). Time of sample explained less than 0.1% of the variance in the (time) adjusted testosterone values at each visit, indicating that the modeling to remove effects of circadian rhythm was successful.

Table 1 provides descriptive statistics for the distributions of age, time at venipuncture, and concentrations of unadjusted and adjusted total testosterone, SHBG, free testosterone and bioavailable testosterone.

### Published testosterone values

Table 2 reports the pooled data from the published reports. On average, there is little testosterone (<1 nmol/L) found in males before the age of 10 years, at which point the levels begin to rise. Between 10 and 15 years of age, the plasma testosterone levels increase nearly seven-fold. The rate of increase slows as the males reach adult levels (∼15 nmol/L) between age 16 and 17 years.

**Table 2:** Published Values of Total Testosterone (nmol/L).

	Cross-Sectional						Longitudinal				Pooled Values		
	Schnakenburg 1980		Elmlinger 2005		Starka 2008		Lee 1974		Hero 2005				
Age	n	Mean (SD)	n	Mean (SD)	n	Mean (SD)	n	Mean (SD)	n	Mean (SD)	# Studies	n	Weighted Mean (SD)
**6–7**			**18**	0.74 (0.15)	**105**	1.6 (2.8)					**2**	**123**	0.74 (0.15)
**8–9**	**32**	0.54 (0.20)	**25**	0.92 (0.27)	**160**	1.3 (2.5)					**3**	**217**	0.68 (0.16)
**10–11**	**45**	2.07 (1.22)	**23**	2.25 (1.12)	**241**	2.7 (3.7)	**34**	2.43 (0.76)	**60**	1.04 (1.39)	**5**	**403**	2.14 (0.51)
**12–13**	**74**	8.18 (4.91)	**40**	10.61 (3.18)	**447**	5.7 (6.8)	**102**	9.96 (2.08)	**118**	5.17 (2.09)	**5**	**781**	8.03 (1.27)
**14–15**	**87**	10.06 (5.07)	**48**	14.54 (4.95)	**616**	9.7 (7.4)	**83**	14.60 (1.93)	**56**	9.36 (5.20)	**5**	**890**	13.46 (1.57)
**16–17**	**54**	14.99 (5.25)	**29**	17.13 (3.13)	**424**	12.7 (7.4)	**12**	18.68 (4.91)	**19**	20.11 (7.63)	**5**	**538**	16.93 (2.16)
**18–19**	**56**	18.10 (3.99)	**23**	15.35 (4.90)	**302**	14.5 (8.2)					**3**	**381**	16.70 (2.89)

Note – Two year intervals reported in the age column (6–7 refers to participants that are between 6.00–7.99 years).

These values are similar to those obtained from the ALSPAC sample (Table 1). Table 3 reports the pooled data after incorporating the adjusted total testosterone values from ALSPAC.

**Table 3:** Pooled Values of Total Testosterone (nmol/L; five published studies plus the new ALSPAC data reported in this communication).

Age	# Studies	n	Weighted Mean (SD)
**6–7**	2	123	0.74 (0.15)
**8–9**	4	556	0.78 (0.08)
**10–11**	6	944	2.00 (0.46)
**12–13**	6	1164	8.04 (1.23)
**14–15**	6	1406	13.58 (1.43)
**16–17**	6	900	16.72 (1.68)
**18–19**	4	442	16.84 (1.97)

### ALSPAC: Growth Spurt and Characteristics of Testosterone Trajectories

Eight participants with very high APHV estimates (>200 months) were excluded since these values are erroneous extrapolations of the model used to estimate APHV, as described previously [19]. The mean age of growth spurt, defined as APHV, is 159.83 months (SD: 13.33 months), with a range of 120 to 189 months (10 to 15.8 years).

The distribution of Age at Peak Testosterone Change in our sample is bimodal (see Discussion), with peaks at 153 and 174 months (Mean: 162.87, SD: 10.42). On the other hand, values of the Peak Testosterone Change are normally distributed (Mean: 0.63 [(nmol/L)/month], SD: 0.21).

### Measure of Average Exposure to Testosterone

We excluded the participants missing two blood samples (n = 68) as their calculated values on this measure were lower compared with participants missing one or none. Values from participants missing one sample were not different from participants with all five blood samples. The values within the subgroup of participants missing one sample, however, varied as a function of the visit with a missing sample (*n* = 213; *r^2^* = 0.08; *p* = 0.002). In line with age-related increases in testosterone, participants missing a sample from the second or third visits had higher estimates, whereas those missing a sample from the fourth or fifth visit had lower estimates. We used residuals from this model to estimate corrected values (mean+residual) in this subgroup. A total of 445 participants had a measure of average exposure to testosterone calculated (Mean: 8.63 nmol/L; SD: 2.15 nmol/L; Range: 3.34–19.50 nmol/L). To determine if replacing undetectable values of testosterone with the assay’s lower limit of sensitivity (see methods) could have an effect on the results, we replaced undetectable values with 0.01 nmol/L (in lieu of 0.28 nmol/L) and recalculated the measure of average exposure to testosterone. A Pearson’s correlation between values from the two calculations shows the variable remains essentially unchanged (r = 0.995, p<0.001).

**Figure 1:**
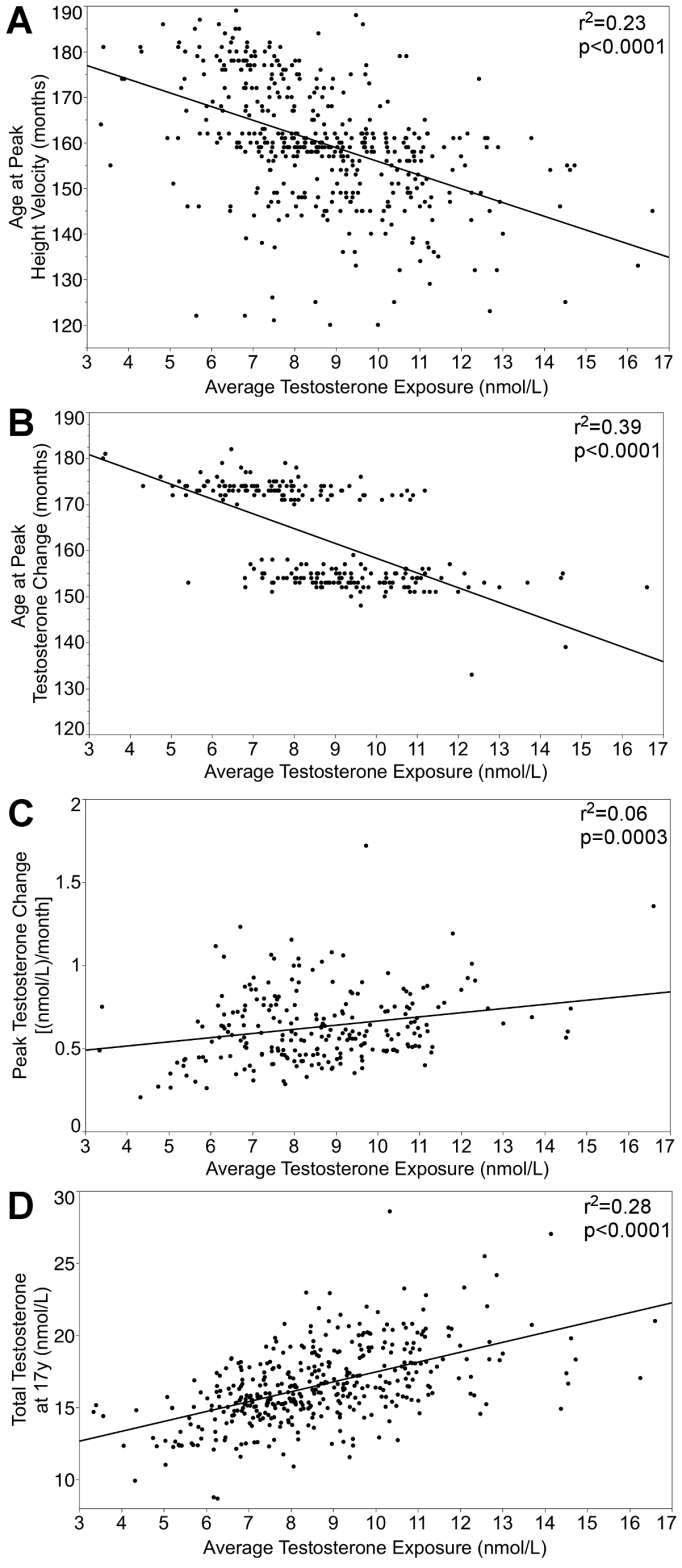
Correlates of the average testosterone exposure. Average testosterone exposure is inversely related to (A) timing of growth spurt and (B) timing of the largest testosterone increase, but is positively related to (C) magnitude of the largest testosterone increase and (D) total testosterone at 17 years.

The average exposure to testosterone is associated with: (A) Age at Peak Height Velocity (*n* = 437; *r^2^* = 0.23; b = −3.01; t = −11.28; *p*<.0001); (B) Age at Peak Testosterone Change (*n* = 232; *r^2^* = 0.39; b = −3.21; t = −12.03; *p*<.0001); (C) Peak Testosterone Change (*n* = 232; *r^2^* = 0.06; b = 0.03; t = 3.68; *p* = .0003); (D) Testosterone at the 17 year visit (*n* = 409; *r^2^* = 0.28; b = 0.68; t = 12.73; *p*<.0001). These associations are shown in Figure 1. Given the bimodal distribution of Age at Peak Testosterone Change, participants were split into two groups on this variable using 160 months as a cut-off point and the analysis carried out with linear regression and shown in Figure 1 (B) was repeated using a one-way ANOVA (n = 232; r^2^ = 0.32; t = −10.42; p<.0001). Note that the analyses related to Peak Testosterone Change (B, C) were restricted to individuals with all five samples, hence the lower sample size.

The means (and SDs) of the quintiles of average exposure to testosterone were: 1^st^ quintile: 5.92 (0.91); 2^nd^: 7.43 (0.29); 3^rd^: 8.43 (0.30); 4^th^ 9.59 (0.39); and 5^th^: 11.72 (1.56) nmol/L.

**Figure 2:**
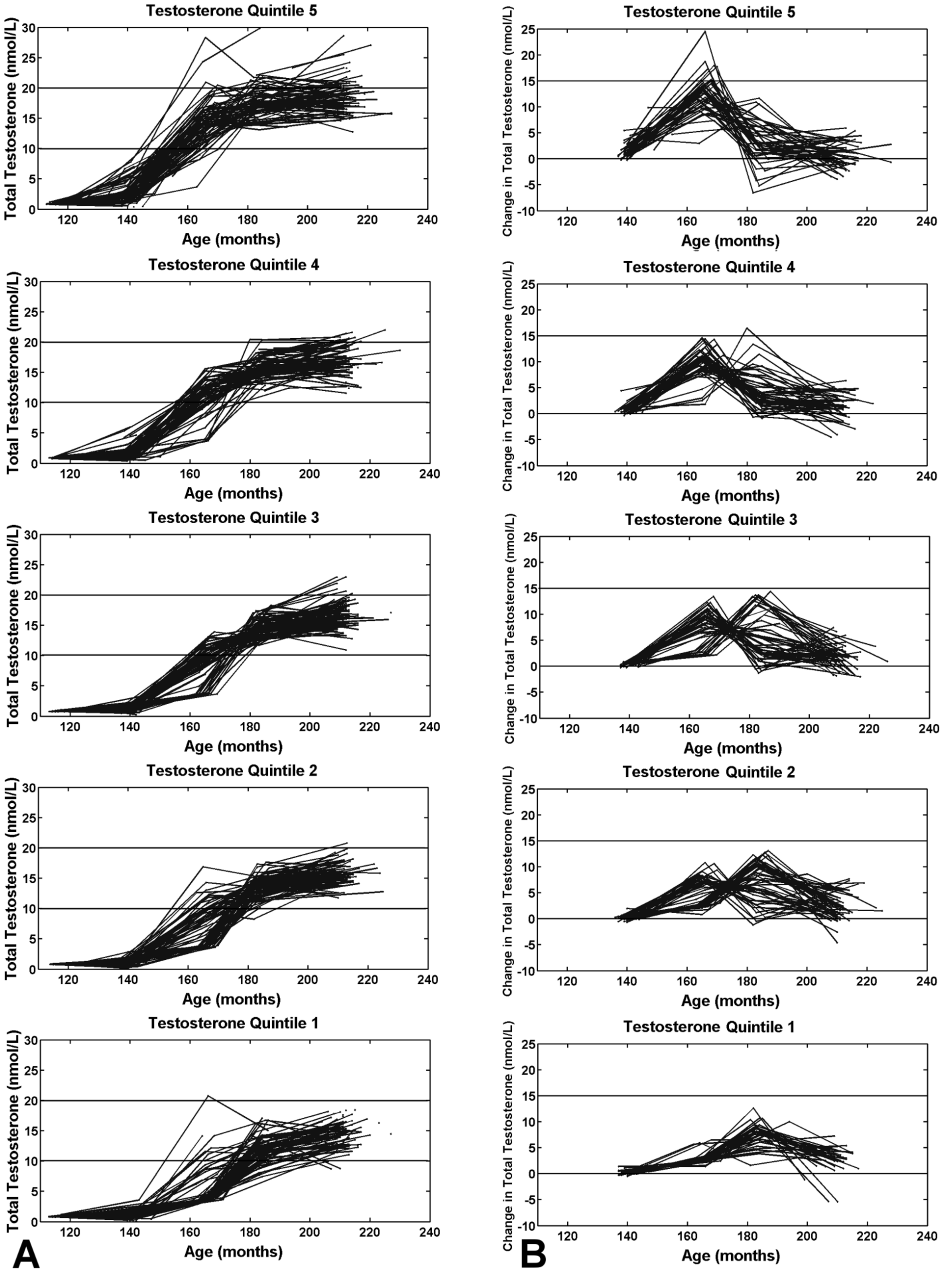
Testosterone trajectories by quintiles of the average testosterone exposure. Higher (Quintile 5) versus lower (Quintile 1) average exposure to testosterone is associated with (A) earlier rising testosterone trajectories that remain relatively high, and (B) earlier onset and greater magnitude of peak change in testosterone. Trajectories are plotted with adjusted testosterone values (see Methods).

Figure 2A shows participants’ testosterone trajectories, while 2B shows testosterone-change trajectories (calculated as Testosterone at Present Visit – Testosterone at Previous Visit) for each of the average-testosterone quintiles. There are clear differences in the shape of these trajectories across the average-testosterone quintiles: participants in the fifth quintile demonstrate a rise in testosterone relatively earlier and of greater magnitude whereas those within the first quintile tend to experience a later and lower increase in testosterone. A notable feature of the testosterone-change trajectories is the peak, indicating when a participant’s testosterone increased the most. Almost all participants from Quintile 5 experience an increase in testosterone-change before age 14 years, whereas the participants in Quintile 1 experience their increase almost exclusively after age 14 years.

**Figure 3:**
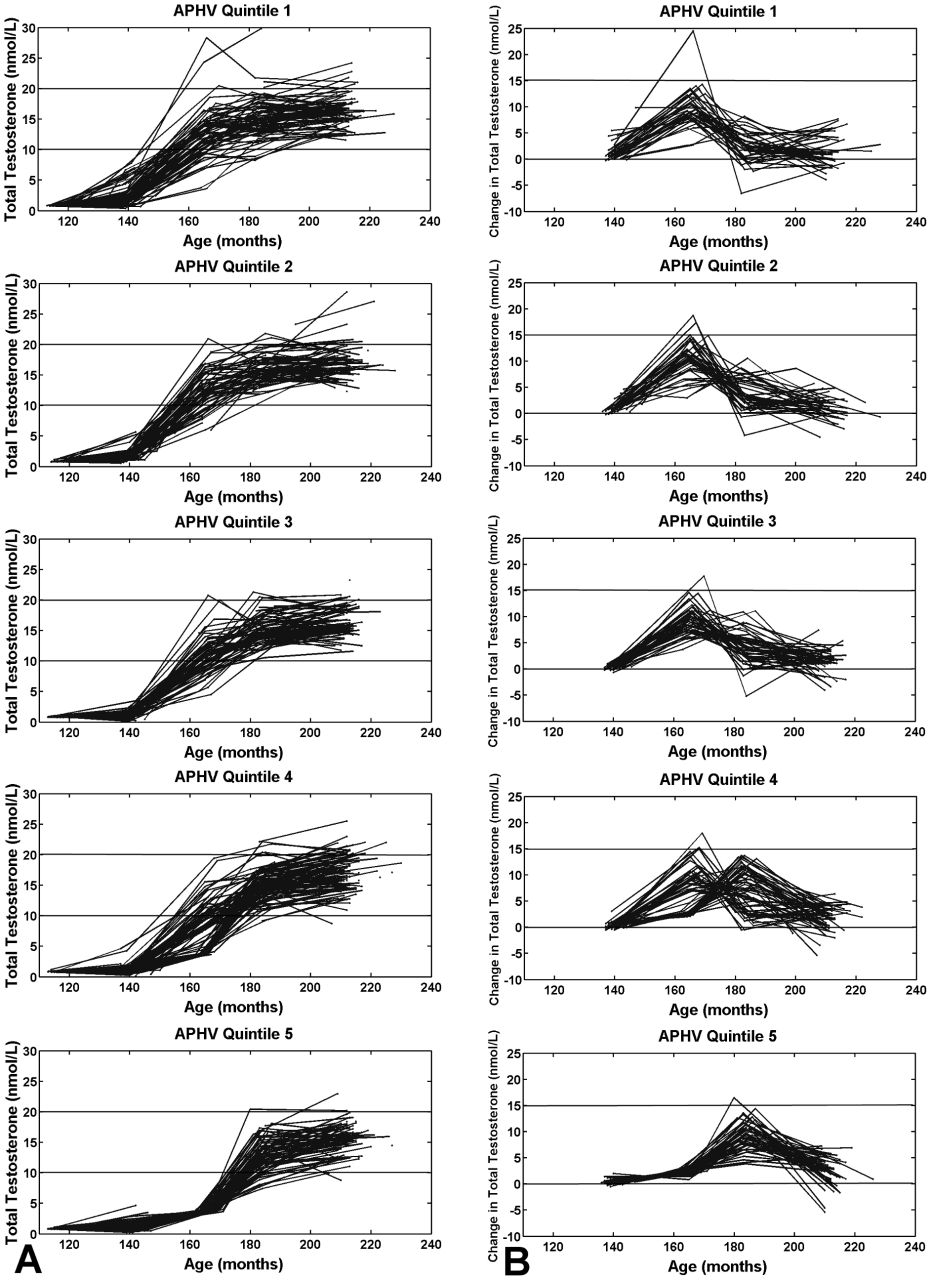
Testosterone trajectories by quintiles of the growth-spurt timing. Earlier (Quintile 1) versus late (Quintile 5) growth spurt is associated with (A) earlier rising testosterone trajectories, and (B) earlier onset of peak change in testosterone. Trajectories are plotted with adjusted testosterone values (see Methods).

Figure 3A shows testosterone trajectories by APHV quintiles, and 3B the testosterone-change trajectories. We find early rising and steep trajectories for early maturers (APHV Quintile 1), and conversely, late rising and gentler trajectories for late maturers (APHV Quintile 5). Similarly to what was seen in Figure 2B, participants in APHV Quintile 1 experience an increase before age 14 years, whereas those in APHV Quintile 5 experience their increase after age 14 years. Again, note that the change trajectories in Figure 2B and 3B show only the 232 participants with all five blood samples.

## Discussion

The quantification of testosterone in male ALSPAC participants yield values that match with ranges reported in other studies. Comparing Table 1 and Table 2, we see similar trends in the mean values of total testosterone between 9 and 19 years of age. Of the five published studies included here, the ALSPAC data resemble the most [11], [14] values reported in the two studies by Elmlinger and Lee. Other studies report slightly lower values in their samples between the ages of 12 to 15 years. The values across studies may vary due to the variance in sample ages within the two year intervals reported, as well as other factors such as ethnicities of the participants included in a given sample [26]. Our synthesized results combining the literature with ALSPAC may serve as useful reference ranges for future investigations that pertain to testosterone in typically developing male adolescents (Table 3).

We also describe a simple measure for characterizing the average exposure to testosterone over adolescence based on longitudinal measures. We use an integral to derive this measure instead of the arithmetic mean; the latter would bias the estimates if the samples were clustered towards the beginning or end of adolescence. Using the integral of a fitted spline helps minimize this bias.

This measure is associated with timing of the pubertal growth spurt and several characteristics of testosterone trajectories: the age and magnitude of the rise in testosterone, and testosterone levels at the end of the trajectory. Participants who achieve an early growth spurt, which is known to be associated with earlier activation of the HPG axis [27], tend to show higher averaged exposure to testosterone during puberty. Similarly, earlier and larger rises in testosterone levels, as well as high levels of testosterone at 17 years are all characteristics of trajectories associated with higher averaged exposure to testosterone over the period defined by the trajectory. Since testosterone levels remain high once they accelerate, early and large increases in testosterone result in a greater exposure to testosterone over puberty. These relationships are demonstrated in Figure 1.

Differences in the shape of testosterone trajectories depicted in Figures 2 and 3 become apparent between 160 to 180 months indicating that possible differences in the timing of the HPG axis activation emerge during this period. This is consistent with the known variation in pubertal timing that is seen across the population [28]. Note that the distribution of Age at Peak Testosterone Change is bimodal likely because our blood samples are taken at two-year intervals. We predict that this distribution would be closer to normal if the frequency of sampling was greater.

Studying testosterone trajectories in a large sample is made challenging by circadian rhythms that cause fluctuations in hormone levels over the time course of a day [18], [29]. As such, the time of blood draw is an important consideration in these studies. In blood collected every 45 minutes over a course of 25.5 hours (in five healthy males), total testosterone peaked at approximately 0500 h and 1000 h, and troughed at approximately 1400 h and 2100 h [30]. The ALSPAC blood samples were obtained throughout the day and time of collection was not standardized across participants and visits, thus requiring statistical adjustment to minimize effects of the circadian rhythm on the hormone values. This was particularly the case in data from the third visit (Teen Focus 2; ∼13 years) where a greater effect of the circadian rhythm was observed; during this visit, time of sampling ranged from 0900 h to 1900 h. The two preceding visits also collected blood samples throughout the day, but the data were not strongly affected by the circadian rhythm given the low testosterone values present in participants at these young ages. The data from visits at ages 15 and 17 years are not affected as much, likely because nearly all the blood samples were taken before the 1400 h nadir. In the first three visits, where many participants were measured after this nadir, the time adjustment increased their values and therefore increased the mean total testosterone for these visits. In comparison, the last two visits have a proportionally smaller adjustment that decreased the mean testosterone slightly. Ideally, all participants in the study would have been sampled at the same time of day, preferably in the morning hours, but this was not possible for logistical reasons. Another decision based on cost-effectiveness in a population-based study is the use of enzyme-linked immunosorbent assays for hormone quantification. This limits detection of very low levels of testosterone and poses challenges when studying pre-pubertal boys.

Testosterone, particularly its bioavailable fraction, is an important androgen that exerts an effect on multiple biological targets in the body. In addition to the multitude of physical changes initiated during puberty, bioavailable testosterone has been implicated in development and maturation of the adolescent brain [9], [10], as well as in psychopathology such as depression and risk of psychosis [31], [32]. Investigations into the effects of hormones have usually been conducted in a cross-sectional manner with venipuncture to collect blood for hormone quantification carried out in temporal proximity to measurement of the outcome of interest (e.g., body composition or behavior). Inquiry into the effect of testosterone exposure over a long time period has rarely been conducted.

We plan to embark on such studies using data in ALSPAC, where the database includes longitudinally collected blood samples and measures of psychopathology throughout adolescence, in addition to a single time-point of Magnetic Resonance Imaging in late adolescence to characterize brain structure and function. In such a design, longitudinal measures can be collapsed into a measure of average exposure and then related to an outcome measured at the end of the study as has been done elsewhere, for example, in the context of air pollution and cardiopulmonary mortality [33]. Our measure of testosterone exposure over adolescence showed associations with salient characteristics of testosterone trajectories. In this way, we can study the effect of testosterone dynamics on shaping the adolescent brain, and then examine the relationship between testosterone and risk of psychopathology. Other investigations aiming to examine longitudinal effects of hormones may find utility in the measure we describe, shown to be associated with characteristics of the hormone trajectory.

## Supporting Information

Table S1 Comparing the sub-sample with the rest of the ALSPAC males.
(DOC)
