# Supplementary material for: Testosterone Trajectories and Reference Ranges in a Large Longitudinal Sample of Male Adolescents
Source: PLoS One. 2014 Sep 30;9(9):e108838. doi: 10.1371/journal.pone.0108838 (PMC4182562; doi:10.1371/journal.pone.0108838)
Supplement: Table S1 — Comparing the sub-sample with the rest of the ALSPAC males. (DOC) [file pone.0108838.s001.doc]

Supplementary Table S1: Comparing the sub-sample with the rest of the ALSPAC males

|  | **ALSPAC males (n=7024)** | | **Sub-sample (n=513)** | |  |  |
| --- | --- | --- | --- | --- | --- | --- |
|  | **N** | **Mean (SD)** | **N** | **Mean (SD)** | **p-value*** | **Cohen’s d** |
| **Gestational age (weeks)** | 6732 | 39.3 (2.0) | 488 | 39.4 (1.9) | 0.222 | -0.06 |
| **Mother’s age at delivery (years)** | 6732 | 28.0 (5.0) | 488 | 29.8 (4.4) | <0.001 | -0.38 |
| **Birth weight (grams)** | 6641 | 3437.9 (583.6) | 483 | 3513.3 (560.2) | 0.006 | -0.13 |
| **BMI at age 7** | 3679 | 16.2 (2.0) | 484 | 15.9 (1.6) | 0.030 | 0.11 |
| **BMI at age 9** | 3277 | 17.5 (2.8) | 496 | 17.3 (2.4) | 0.043 | 0.10 |
| **BMI at age 17** | 1742 | 22.7 (4.0) | 472 | 22.2 (3.3) | 0.005 | 0.15 |
| **Verbal IQ at age 8** | 3196 | 106.7 (17.4) | 475 | 113.0 (17.7) | <0.001 | -0.36 |
| **Total IQ at age 8** | 3182 | 103.1 (16.9) | 472 | 110.4 (17.0) | <0.001 | -0.42 |
|  | **N** | **%** | **N** | **%** | **Chi-2 p-value** |  |
| **Mothers’ marital status at child’s birth** | 6281 |  | 482 |  | <0.001 |  |
| Never married | 1252 | 19.9 | 50 | 10.4 |  |  |
| Ever married | 5029 | 80.1 | 432 | 89.6 |  |  |
| **Mothers’ smoking status at 18 weeks gestation** | 6256 |  | 479 |  | <0.001 |  |
| Never smoked | 2993 | 47.8 | 299 | 62.4 |  |  |
| Ever smoked | 3263 | 52.2 | 180 | 37.6 |  |  |
| **Maternal social class** | 4748 |  | 434 |  | <0.001 |  |
| Manual | 973 | 20.5 | 47 | 10.8 |  |  |
| Non-manual | 3775 | 79.5 | 387 | 89.2 |  |  |
| **Paternal social class** | 5172 |  | 452 |  | <0.001 |  |
| Manual | 2347 | 45.4 | 131 | 29.0 |  |  |
| Non-manual | 2825 | 54.6 | 321 | 71.0 |  |  |
| **Maternal education** | 5934 |  | 477 |  | <0.001 |  |
| O-level or less | 3957 | 66.7 | 210 | 44.0 |  |  |
| At least A-level | 1977 | 33.3 | 267 | 56.0 |  |  |
| **Paternal education** | 5674 |  | 471 |  | <0.001 |  |
| O-level or less | 3245 | 57.2 | 189 | 40.1 |  |  |
| At least A-level | 2429 | 42.8 | 282 | 59.9 |  |  |
| **Ethnicity** | 5749 |  | 476 |  | 0.028 |  |
| White | 5448 | 94.8 | 462 | 97.1 |  |  |
| Non-white | 301 | 5.2 | 14 | 2.9 |  |  |

*from the t-test unless otherwise stated; BMI, Body Mass Index; IQ, Intelligence Quotient; SD, standard deviation
